# Understanding Traditional Chinese Medicine Therapeutics: An Overview of the Basics and Clinical Applications

**DOI:** 10.3390/healthcare9030257

**Published:** 2021-03-01

**Authors:** Luís Carlos Matos, Jorge Pereira Machado, Fernando Jorge Monteiro, Henry Johannes Greten

**Affiliations:** 1 Faculdade de Engenharia da Universidade do Porto, 4200-465 Porto, Portugal; fjmont@fe.up.pt; 2 CBSIn—Centro de Biociências em Saúde Integrativa, Atlântico Business School, 4405-604 Vila Nova de Gaia, Portugal; jmachado@icbas.up.pt; 3 CTEC—Centro Transdisciplinar de Estudos da Consciência, Universidade Fernando Pessoa, 4249-004 Porto, Portugal; 4 ICBAS—Institute of Biomedical Sciences Abel Salazar, University of Porto, 4050-313 Porto, Portugal; heidelbergschool@aol.com; 5 INEB—Instituto de Engenharia Biomédica, Universidade do Porto, 4200-135 Porto, Portugal; 6 German Society of Traditional Chinese Medicine, 69126 Heidelberg, Germany

**Keywords:** traditional Chinese medicine, herbs, dietetics, acupuncture, moxibustion, “Tuina”, “Qigong”, “Taijiquan”, Heidelberg Model of TCM

## Abstract

Traditional Chinese medicine (TCM) is a systematic healthcare system developed from clinical experience based on a scientific model of regulation. TCM relies on unique theories and practices to treat diseases and enhance health. These practices include Chinese herbal medicine and dietetics, acupuncture and moxibustion, and other non-medication therapies such as Chinese bodywork or manual therapy, known as “Tuina”, and traditional biofeedback exercises, known as “Qigong” and “Taijiquan”. The integration of TCM in Western health systems and research requires a rational communicable theory, scientific proof of efficacy and safety, and quality control measures. Understanding the structural concepts of the TCM language makes possible the parallelism to Western physiology, and the inherent rational use of the reflex therapeutic systems, anti-inflammatory mechanisms and mental training involved, for example, in acupuncture and “Qigong”. The results of TCM clinical trials and fundamental research on its nature and mechanisms have encouraged the development and application of well-designed research strategies such as double blinding in acupucture to overcome limitations and resistances in integrating these practices into the existing biomedical paradigms of the West. This review aims to overview some TCM theoretical concepts and the evidence-based clinical application of TCM’s leading practices to create an easy-to-consult and condensed source of information, available for the healthcare community, facilitating the understanding and communication between conventional health professionals and TCM practitioners and acupuncturists.

## 1. Introduction

Although the origin of TCM remains uncertain, some evidence points to more than 5000 years of history. Indeed, some archaeological findings of acupuncture needles and traces of herbal treatments suggest 4000 to 8000 years of existence [1,2]. The “Yijing” (“I Ching” or The Book of Changes) and the “Huangdi Neijing” (The Yellow Emperor’s Classic of Internal Medicine) are the oldest known written sources of information in TCM philosophy and clinical application. The “Yijing”, dating back 3000 to 5000 years, describes the course of life systematically based on a mathematical model of regulation. It describes its changes and modalities, offering advice on personal emotional lifestyle and guidance [3,4], while the “Huangdi Neijing” is comparable in importance to the Hippocratic Corpus in Greek medicine [5].

The main TCM theories include the teaching of “yin” and “yang” and the Five trespasses Phases (“Elements”). They describe the activity of effects and functional powers involved in body function such as the “qi”, the “blood” or “xue”, as well as the effects of active and resting fluids “jin ye”, and the differential diagnosis of syndromes, while the primary practices include acupuncture and moxibustion, the use of Chinese herbs and dietetics, and “Tuina”, “Qigong” and “Taijiquan”, commonly known as Tai Chi. Ancient Chinese physicians postulated that everything is made of the same “substance”, the “qi”. This philosophy stands for oneness and wholeness as part of the same paradigm, considering that all existing things are symbiotically connected through the system of “qi” [6]. One of the main goals of TCM is to balance the effects of the body’s “qi”, known in the West as the “Vital Force”, to live in harmony with the surrounding “qi”. According to ancient Chinese culture, this includes the energies of “Heaven”, such as the energy of the sun, moon, planets, and constellations, and the energies of “Earth”, such as the effects of geographical location, the energy of the plants, soil, water, animals, and natural formations [6]. TCM aims to treat not only the secondary manifestations (“biao”), but also the primary causes (“ben”) of several chronic and acute conditions; for example, in internal medicine, gynecology, pediatrics, traumatology, external medicine, dermatology, emergency medicine, and eye, ear, nose and throat treatments.

**Figure 1:**
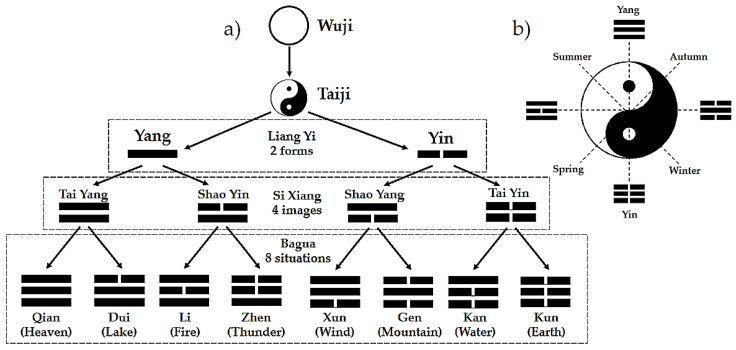
The creation of “yin yang” and “Bagua” (**a**); the “Taiji” symbol (**b**).

The “Yijing” analysis performed by the mathematician Gottfried Leibniz in 1697 [4] allows for understanding TCM as a logical model of a system’s biology with a structural mathematical language. The analogy to the binary system, considering 0 as “yin” and 1 as “yang”, allows for the understanding of monograms, bigrams and trigrams, which are related to the Phases and the circular regulatory processes seen, for example, in rhythmical seasonal changes, daytime processes and human behavior (Figure 1a,b) [7,8]. These circular processes are expressed as sinusoidal functions, which integrate the concepts of “yin”, “yang”, and the Phases as cybernetic elements and vegetative functional tendencies in human physiological regulation.

The “yin yang” concept is central in TCM philosophy. This pair of terms is used to describe functional relationships in Chinese culture and language and, metaphorically, has a broad list of possible meanings. Common examples related to “yin” are cold, female, earth, water, weak, dark, night, winter, passive, and interior, while those related to “yang” are hot, male, heaven, fire, strong, light, day, summer, active and exterior [9]. Within the regulatory (cybernetic) context, “yin” and “yang” may enclose the meanings shown in Table 1.

The Five Phases, often referred to as Five Elements (“Wu Xing”), are Wood, Fire, Earth, Metal, and Water. The four bigrams shown in Figure 1a, created from the monograms “yin yang”, represent the Phases. Each one of these bigrams gives rise to two trigrams, representing the “orbs” or signs.

Therefore, it is possible to access a person’s functional state by the visible signs and symptoms, and relate this to the cycle’s correspondent part. This structure might be used in different knowledge areas, such as anatomy, physiology, and human psychology [9].

Within the medical context, these terms are used to describe the functional concepts shown in Table 2.

Traditionally, the Five Phases influence each other both physiologically (“sheng” and “ke” cycle) and pathologically (“cheng”, “wu” and, to some extent, the “sheng” cycle). If the Five Phases’ balance is disturbed, it is more likely that pathological syndromes will manifest themselves [11].

**Figure 2:**
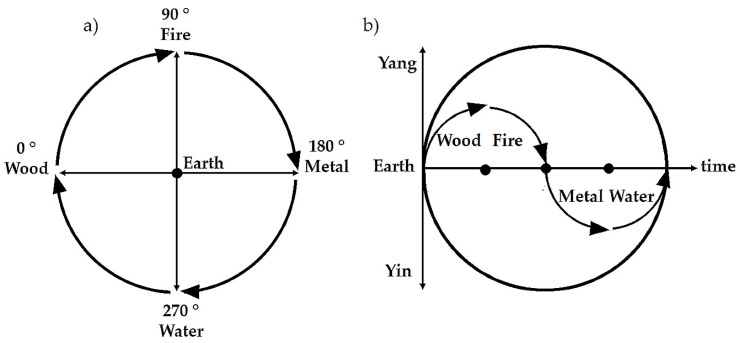
Compass Rose (**a**) and circular function of vegetative regulation (**b**) with the “yang” (upregulation) and “yin” (downregulation) components and respective Phases.

In the Compass Rose cycle, the Earth Phase is considered the center, the baseline or target value, around which upregulation (Wood and Fire) and downregulation (Metal and Water) processes are established (Figure 2a). The Compass Rose dynamics can be represented as a circular function of vegetative regulation, with “yin” and “yang” components, wherein the abscissa axis represents the Earth Phase (Figure 2b).

The functional activity related to each Phase can be metaphorically described as follows: Wood is the creation of potential; Fire is the transformation of potential into function; Metal shows a relative lack of energy, as well as the rhythmical distribution of energy; Water is regeneration; Earth stimulates transformation and evolution. These main actions are related to the overall vegetative activity, ruled by transmitters and neuronal pathways, whereby sympathetic functions are more active in “yang” phases, and in the “yin” phases, parasympathetic (vagal) activity is relatively more present [3].

The vegetative system integrates the sympathetic, parasympathetic, and enteric nervous systems involved in the regulation cycle. For example, in stress situations, the enteric nervous system is less active; otherwise, defecation would be dysfunctional. On the other hand, in the downregulation phases or even in Fire and Metal, the enteric nervous system is more active in restoring the energy levels. This system of functional regulation can also integrate muscle tonus and motion patterns by hypertonic, hyperdynamic, hypotonic, and hypodynamic functional patterns, or the RAAS (renin–angiotensin–aldosterone system), which is more active in the “yang” phases, above the target value, and less active below, in the “yin” phases domain [3,7,10,12,13].

In TCM, physiological functions, pathological changes, and the relationship of an organ with the fundamental substances (“qi”, “xue”, “jin ye”), other organs, and other parts of the body are more important than its anatomical structure. In Western medicine, anatomical structure plays an essential role in the process of “judging the exterior from the interior”. TCM chooses the pattern of “judging the interior from the exterior” by observing the body’s outward appearance and changes, considering that what occurs inside will manifest itself on the body’s surface [14].

In Western medicine, symptom-perception gradually becomes disease-perception, which means a deeper understanding of its nature. In TCM, the manifestation and differentiation of syndromes rather than diseases are essential to selecting the appropriate therapeutic intervention. Syndromes, also known as “zheng”, are symptoms of disturbances of visceral function and “yin yang” balance evoked by pathogenic factors. These originate signs that can be detected, for example, in tongue and pulse diagnosis. Many Western medicine diseases may be similar to some “syndromes” in TCM, while some “syndromes” may include certain Western medicine diseases [14]. For example, the kidney’s essence, also known as “jing”, is an essential concept related to the process of aging. According to TCM, each person is born with a finite amount of “jing” stored in the kidneys and gradually uses it through life. Some authors consider “kidney deficiency syndrome” equivalent to aging in Western medicine. Similarly, “heart qi deficiency” is associated with cardiac insufficiency [15].

The term “zang-fu” in Chinese refers not only to the anatomical entities of the internal organs, but also to a generalization of the human body’s function. TCM divides the internal organs into two groups, typically coupled in “yin yang” pairs per Phase: “Zang”, or solid organs, which are considered “yin” organs (heart and pericardium, liver, spleen, lung, and kidney), and “fu” or hollow organs, which are considered “yang” organs (small intestine and triple burner, gall bladder, stomach, large intestine, and bladder) [16]. Within the interstitial connective tissue, TCM meridians or conduits and collaterals constitute a network called “jing luo”. These conduits, believed to serve as channels for the flow of “qi” and “xue”, are connections between acupoints with effects on specific orbs’ clinical signs. There are 12 primary meridians, 6 “yin” and six “yang”, classified as the 3 “yang” meridians of the hand, the 3 “yang” meridians of the foot, 3 “yin” meridians of the hand, and 3 “yin” meridians of the foot. The “yin” meridians pertain to the “zang” organs and the “yang” meridians to the “fu” organs. In this way, “qi” and “xue” flow through them and reach every part of the body in a cyclical circulation [17,18].

## 2. Chinese Herbal Medicine and Dietetics

Although the written history of Chinese materia medica dates back from the Eastern Han dynasty (AD 25–220), with the “Shen Nong Ben Cao Jing” (Divine Husbandman’s Classic of Materia Medica), also known as The Canon of Materia Medica, or Shennong’s Herbal Classic [19], Chinese knowledge of botanic medicine dates back to the discovery of “Ma Huang” (*herba ephedrae*), around 3000 BC. This herb was initially used as a stimulant but was also used for respiratory disorders and other diseases. The active ingredient in *herba ephedrae* is ephedrine, an effective bronchodilator, making “Ma Huang” central in asthma treatment [20].

Chinese herbal medicine and dietetics, also known as Chinese materia medica, follow the same diagnostic principles as acupuncture, “Tuina” and “Qigong”. This branch of TCM is focused on the beneficial effects of herbs and foods on the body. Those are classified according to the thermal nature, flavor or “sapor”, organ network, and functional effect direction. The thermal nature has a cooling or warming effect on the body by acting on microcirculation. The “sapor” relates to the Five Phases and regulates a particular organ network [21].

Regarding the thermal nature, “hot” herbs and foods such as pepper, chili, and garlic have heating, warming effects on the body, increasing “yang”, speeding up the “qi”, activating, dispersing, and moving upward and outward, warming the bowels and viscera (“zang-fu”), eliminating external and internal cold, and mobilizing defense energy. Oppositely, “Cold” foods, such as tomatoes and bananas, cool the body, cool internal heat, and have a calming effect on the spirit (“shen”). Warm and cool herbs and foods have a milder effect on the body, strengthening “yang” and “qi” and supplementing body fluids (“jin ye”) and “xue”, respectively [22].

According to the Heidelberg model of TCM, the flavors or “sapors” are considered therapeutic vectors within the system described in Figure 2 [21]. Flavors can be classified according to their “yin” or “yang” quality, and each one belongs to one of the Five Phases:

- Sweet belongs to the Phase Earth (spleen and stomach). It has a warming, strengthening, harmonizing, relaxing, and moistening effect. Sweet has the most potent supplementing effect on the body;
- Acrid or pungent belongs to the Phase Metal (lung and large intestine). It moves the “qi”, invigorates energy circulation, loosens stagnation, disperses, opens pores, frees the surface of exogenous disease factors, and produces perspiration;
- Salty belongs to the Phase Water (kidney and bladder). It cools, moistens, produces a downward bearing, softens, and loosens;
- Sour belongs to the Phase Wood (liver and gallbladder). It has an astringent effect, and gathers and preserves fluids;
- Bitter belongs to the Phase Fire (heart and small intestine). It has a drying, hardening, and downward-bearing effect.

Along with their general effects, each “sapor” directly affects a specific “orb” within each Phase and has an indirect effect within the Phases network. As mentioned in the “Huangdi Neijing”, “Sourness enters the liver, bitterness enters the heart, sweetness enters the spleen, acridity enters the lungs, and saltiness enters the kidneys.” Thus, “Sourness enters the sinews, bitterness enters the blood, acridity enters the qi, sweetness enters the flesh, and saltiness enters the bones.”

**Figure 3:**
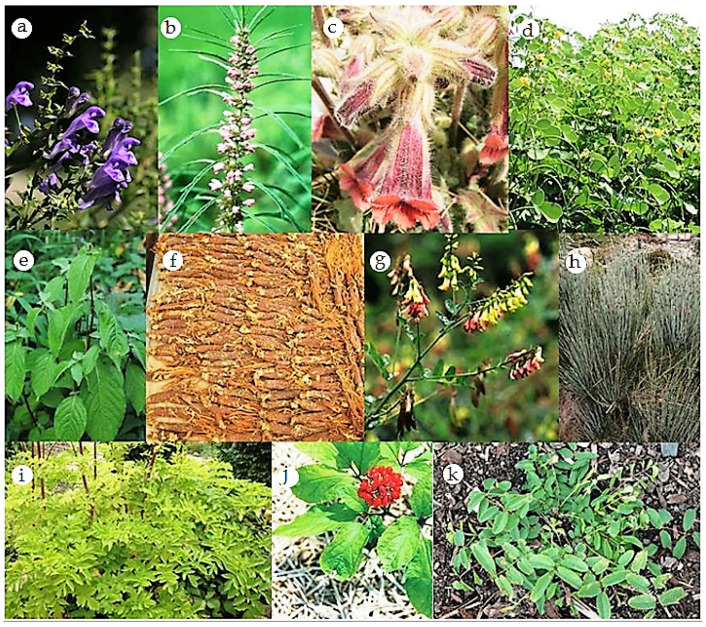
Examples of herbs used in Chinese herbal medicine: (**a**) *Scutellaria*; (**b**) *Leonurus artemisia*; (**c**) *Rehmannia*; (**d**) *Cassia obtusifolia*; (**e**) *Achyranthis bidentata*; (**f**) Panax ginseng root; (**g**) *Astragalus membranaceus*; (**h**) *Ephedra intermedia*; (**i**) *Angelica sinensis*; (**j**) Panax ginseng plant; (**k**) *Sanguisorba officinalis*. Reprinted with permission from ref. [23]. Copyright 2019 Jade Institute (Seattle, WA, USA).

Herbs and foods can also be classified according to the effective therapeutic direction. Therefore, they can act on the surface or “extima”, or affect the depth, the “intima”, or the “yin” itself [21]. Some of the selected plants used in TCM are shown in Figure 3.

The plants listed above, as an example, are frequently used in the form of infusions, mixed with others in predefined proportions, giving rise to specific formulas or decoctions directed to the treatment of certain diseases and conditions. The roots are the most commonly used part; however, in some formulas, the leaves and stems are also used. The preparation of decoctions is an art, and its application’s success is intimately related to an accurate diagnosis.

### Chinese Herbal Medicine Tendencies in Research and Clinical Applications

Herbal treatment is among the most commonly used of the complementary and alternative medicine (CAM) therapies, and many of the used herbs originate from TCM. The popularity of and interest in CAM have increased in Western societies. Socioeconomic, demographic, and health indicators seem to be related to the prevalence of CAM in each country [24]. In 2002, 2007, and 2012, the percentages of American adults who used any CAM approach in the past 12 months were 32.3%, 35.5%, and 33.2%, respectively [25]. The variability of CAM’s prevalence in different countries is significant. In France and Germany, two European countries with a higher prevalence, those values reached 49% and 46%, respectively [24], while in Australia it was about 52% [26], and in Singapore nearly 76% [27]. Although the statistics correspond to different years, the relative global analysis remains accurate, showing a significant variability.

Several herbs used in Chinese herbal medicine have been used in Western medicine. For example, the ginseng decoction used to invigorate “qi” and prevent prostration can be prescribed in hemorrhagic or cardiogenic shock [15]. Artemisia annua, also known as sweet wormwood, became popular in the West after Tu Youyou was awarded the 2015 Nobel Prize in Physiology or Medicine for her discoveries concerning a novel therapy against Malaria. Tu Youyou, a pharmacologist trained in TCM, start working in the Institute of Chinese Materia Medica of the Academy of Traditional Chinese Medicine (Beijing, China) in 1955. In 1969, she started leading a research group focused on searching for antimalarial drugs among traditional Chinese medicines. After reviewing the traditional Chinese medical literature and folk recipes, and interviewing experienced TCM practitioners, a reference to sweet wormwood (“Qinghao”), which had been used in China around 400 AD to treat “intermittent fevers” (a symptom of malaria), came to her attention. The first clinical trial was carried in October 1972, after multiple extraction studies. The trial was successful, and the team started working in the isolation and purification of what came to be known as artemisinin. Later, the team discovered dihydroartemisinin, a derivate of artemisinin that is ten times more potent clinically, demonstrating a high efficacy, rapid action and low toxicity [28].

**Figure 4:**
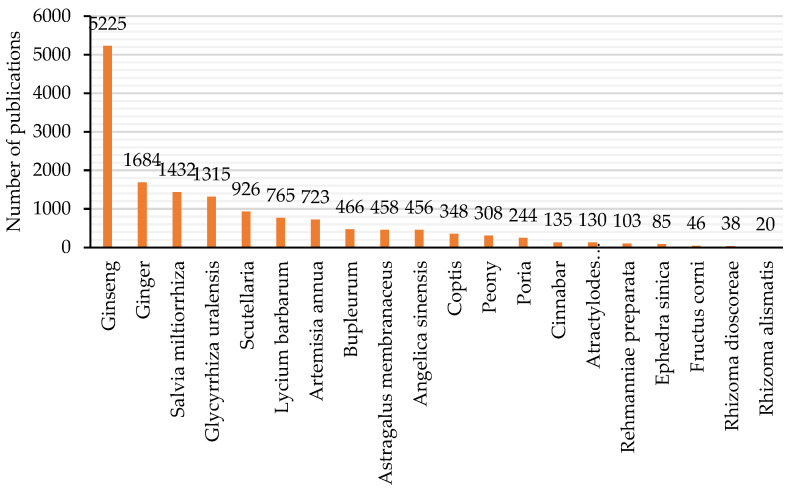
Total number of publications per herb in PubMed, using the article’s title as the searching criteria.

Studying Chinese herbs pharmacology, as well as their action mechanisms, is crucial to generate consensus regarding TCM integration into Western healthcare systems and research. In the last few decades, the number of articles on Chinese herbs published in international scientific journals has increased exponentially [29]; likewise, the trend in published articles on TCM in general has also increased [30]. Indeed, with a simple analysis of PubMed, the previous observation can be quickly confirmed. Therefore, twenty commonly used Chinese herbs were chosen, and using the PubMed database, their names were searched in the titles of articles in general. Figure 4 shows the retrieved numbers of publications per herb (data accessed on 10 February 2021).

**Figure 5:**
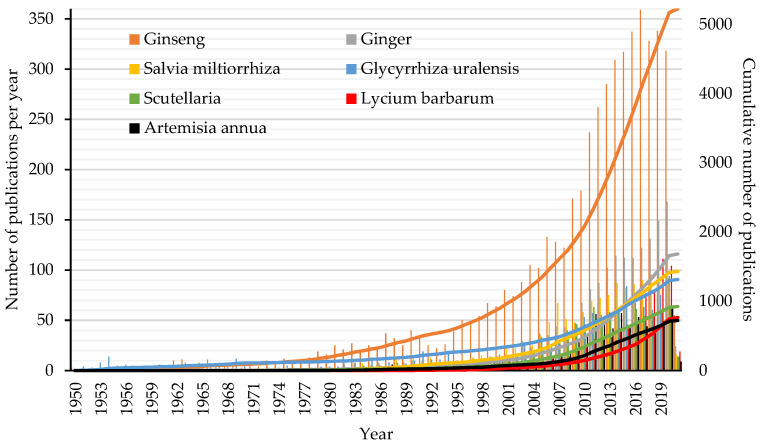
Total number of publications per year (the histograms) and the cumulative number of publications (the continuous lines) since 1950.

Ginseng is the herb that has gathered the most research attention, followed by ginger, *Salvia miltiorrhiza*, *Glycyrrhiza uralensis*, *Scutellaria*, *Lycium barbarum*, and *Artemisia annua*. Considering these seven herbs, Figure 5 shows the total number of publications per year (the histograms) and the cumulative number of publications (the continuous lines) since 1950.

Nowadays, ginseng is an active ingredient in modern medicine, and is often used as a tonic for invigoration and fortification in fatigue, debility, or declining sexual capacity, improving concentration, helping convalescence, and enhancing the immune system. Although clinical trials support these indications, ginseng’s properties had already bene described in Chinese herbal medicine classics.

The pharmacological properties of ginseng were firstly described in Shennong’s Herbal Classic. In this classic of materia medica, ginseng is described as being capable of nourishing or strengthening the five vital organs of the body (the spleen, lung, heart, kidney, and liver), having sedative properties, being used for palpitations to restore a regular pulse, for dispelling pathogenic factors, for improving visual acuity and mental activity, and for enhancing longevity with long-term intake. Later on, in the Mingyi Bielu written by Hongjing Tao (AD 456–536), additional properties can be found, including curing internal coldness, pain in the chest or abdomen, sensations of fullness in the chest, vomiting and diarrhea, relieving thirst and feelings of solidness, enhancing cognitive function, and improving blood circulation. The Bencao Gangmu written by Shizhen Li (1518–1593) describes ginseng as capable of treating general weakness, spontaneous sweating and fever, vertigo and headache, regurgitation and vomiting, alternating fever and chills, chronic diarrhea, increased urination or strangury, fatigue, externally contracted wind or hot attacks, cramps, vomiting blood (hematemesis), bleeding from the rectum, bloody urinary leakage, abnormal uterine bleeding, and discomfort before or after parturition [31].

Chinese herbal medicine is a complicated and vast field of study. While Western medicine seeks to isolate a single active ingredient, herbal medicine relies on the synergistic action of the constituents of a herb or decoction. These combinations may yield a wide variety of effects, such as anti-inflammatory, antioxidative, antipyretic, antidepressant, antimicrobial activity, relaxation of blood vessel walls, skeletal muscle relaxation, and anticonvulsant activity, among many others [32].

Chinese herbal medicine’s clinical effects must be tested in well-controlled conditions using well-designed randomized controlled trials (RCTs). These should be published under a rigorous peer-reviewed process and scrutinized to minimize the effects of methodological flaws, and to create clear evidence of security and efficiency. The highest form of evidence comes from systematic reviews and meta-analyses, and, therefore, to have an idea of the degree of clinical evidence available for Chinese herbal medicine, a simple analysis was performed on 20 February 2021, using the PubMed database. The searching criteria were based on using the combination of terms (Chinese herbal medicine (Title)) AND (systematic review and meta-analysis (Title)) NOT (protocol (Title)) in the titles of the articles, and considering a publication period set between 1 January 2019 and 20 February 2021. Twenty-six articles were obtained using this strategy. Three articles were excluded because one was a commentary, the other was a protocol, and the last used an animal model. The diversity of pathologies covered by the remaining articles is considerable, with three articles related to cancer (involving 54, 14 and 18 RCTs) [33,34,35], three related to hypertension (involving 30, 17 and 39 RCTs) [36,37,38], three related to COVID-19 (involving 19, 7 and 18 RCTs) [39,40,41], two related to depression (involving 40 and 16 RCTs) [42,43], two related to headache (involving 31 and 30 RCTs) [44,45], and the others related to allergic rhinitis (involving 17 RCTs) [46], spinal cord injury (involving 26 RCTs) [47], breast pain (involving 17 RCTs) [48], primary Raynaud’s phenomenon (involving 8 RCTs) [49], insomnia (involving 13 RCTs) [50], erectile dysfunction (involving 11 RCTs) [51], post-stroke spasticity (involving 35 RCTs) [52], irritable bowel syndrome with diarrhea (involving 21 RCTs) [53], diabetic kidney disease (involving 20 RCTs) [54], and Wilson’s disease (involving 18 RCTs) [55]. All articles reported the beneficial effects of Chinese herbal medicine when used as a primary treatment or adjunct therapy to Western medicine. Nevertheless, almost all meta-analyses reported the low quality of the included RCTs, identifying problems in randomization, concealment of allocation, blinding, dropouts, heterogeneity, and sample size.

From the above, the meta-analysis conducted by Wang et al. (2019) included RCTs whose methodological quality was classified as moderate, providing evidence to a certain extent for Chinese herbal medicine’s routine use for depression [42]. In addition, in the study conducted by Shi et al. (2019), despite the clinical heterogeneity, the authors found that when comparing Chinese herbal medicine with a placebo, the overall quality of evidence according to the primary outcome measures was moderate or high, and the overall quality of evidence in Chinese herbal medicine relative to Western medicine was low or moderate. These results support Chinese herbal medicine’s efficiency for use on headache patients, at least to a certain extent [45].

The previous results must be evaluated with caution, as the searching criteria of the analysis mentioned above, given that the combination of terms might have been restrictive and therefore excluded systematic reviews and meta-analyses presenting a higher level of evidence. For example, by using a different conjugation of terms, (Chinese herbs (Title/Abstract)) OR (Chinese herbal medicine (Title/Abstract)) OR (Decoction (Title/Abstract)) AND (systematic review and meta-analysis (Title)) NOT (protocol (Title)), in the same period, 64 articles were retrieved. Those articles covered near 30 different pathologies, among which cancer-related conditions were the most frequent, followed by chronic obstructive pulmonary disease, COVID-19 and hypertension.

According to the World Health Organization (WHO, Geneva, Switzerland), trachea, bronchus and lung cancers are among the top 10 causes of death globally, plus stomach, colon and rectum cancers in upper-middle and high-income countries. Cancer remains a scourge, and despite some advances, often the treatments continue to be, in some cases, painful, damaging, expensive and ineffective. Surgery, radiotherapy and chemotherapy are still the primary treatment methods, often causing unpleasant side effects the severity of which varies from person to person. Here, Chinese herbal medicine can be a promising ally in an integrative strategy based on evidence.

Wang et al. (2019) conducted a systematic review and meta-analysis of high-quality RCTs using Chinese formulas, including “Danshen” for cancer treatment. These authors found that the “Danshen” formulae combined with chemotherapy for cancer treatment were better than a conventional drug treatment plan alone [56]. Indeed, radix Salviae Miltiorrhizae (“Danshen”) has been used in clinical practice for over 2000 years. This herb enhances blood circulation and clears blood stasis, two crucial qualities in cancer treatment.

In 2019, an overview of the RCTs on TCM in cancer care was published in Chinese in the high-ranked journal The Lancet. The authors found that from the 5834 RCTs (involving 477,147 participants), only 62 publications were indexed in MEDLINE. The main three cancers treated with TCM, either combined with conventional treatment or for the treatment of symptoms and side-effects, were lung cancer, stomach cancer, and breast cancer, and the primary outcomes were clinical symptom improvement (3712 RCTs; 63.6%) followed by quality of life (2725 RCTs; 46.7%) and biomarker indices (2384 RCTs; 40.9%). Although further comprehensive evaluations of the beneficial effects and safety of these TCM modalities are needed, the authors concluded that in comparison with conventional treatment, TCM alone or combined with conventional treatment had a better effect in cancer care [57]. Even so, validated standards for this kind of treatment are still lacking, which causes problems as regards oncologists’ acceptance [58].

Despite the increasing demand and positive therapeutic effects, rigorous research on botanicals and Chinese herbal preparations is crucial for their acceptance into mainstream science. The evaluation of toxicological issues, including dose–response curves, the standardization of extract analysis, and a better understanding of neurochemical mechanisms, is critical. Unfortunately, much of these studies are lacking [19,20].

## 3. Acupuncture, Moxibustion, and Cupping

Acu-moxa therapy (“zhen jiu”) is a general term covering several techniques designed to stimulate acupoints located on the body along the circulation tracts or conduits. These practices either alleviate local symptoms, affect orbs’ functions, or treat the underlying imbalance causing the symptoms. Although TCM involves many other techniques, acupuncture and moxibustion are the most popular [59].

### 3.1. Acupuncture

Acupuncture (“zhen”) is based on the proper insertion and manipulation of needles of various gauges and lengths into the skin at specific acupoints. Acupuncture can be traced back to the Stone Age in ancient China, when people used pointed stone implements and pressure to alleviate illness. With the advancement of technology, the stone-needle was replaced with bamboo, and later, metals. Acupuncture treatment is offered in several distinct styles, including Japanese styles, Korean hand acupuncture, Leamington Five-Elements acupuncture, French energetic acupuncture, and Chinese TCM style. There are also specialized approaches, known as microsystems, such as hand–foot acupuncture, and auricular and scalp acupuncture. Although the use of metal needles and moxibustion continues to be the most common technical approach, today, both electricity [60,61] and lasers [60,62,63] are used to replace handheld needles in certain circumstances [64].

**Figure 6:**
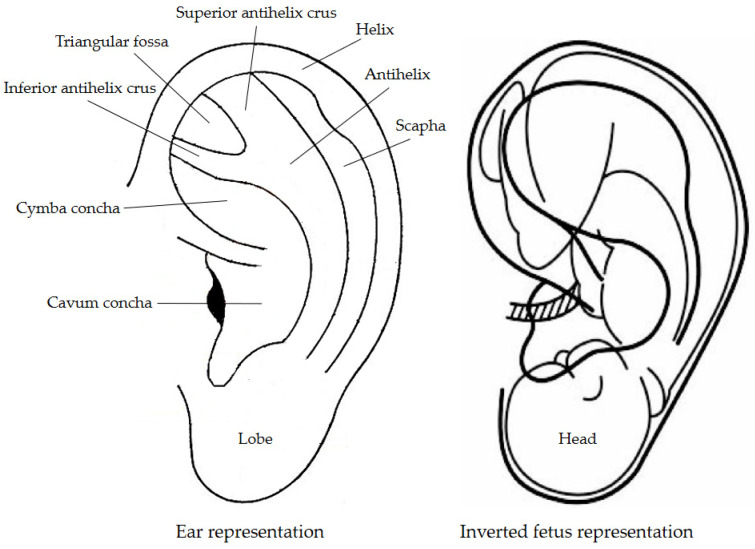
Representation of some regions considered in ear acupuncture and the relation to the inverted fetus representation. Reprinted from ref. [65,66].

Auricular acupuncture (ear acupuncture, auricular therapy) treats pain and certain diseases by placing needles on the external ear’s specific points. The auricular points are distributed in the pattern of an upside-down fetus. The ear lobe is related to the head and face region, upper extremities are in the scapha region, the lower extremities are in the superior antihelix crus region, and the internal organs are located in the cavum and cymba concha areas (Figure 6).

Pathology, whether on a specific organ or within a body system, is reflected in the auricle, which can exhibit external changes such as discoloration, tenderness and decreased electrical resistance in the corresponding meridian pathway within the ear, and even mild morphological changes (scarring) over time [67]. Although there is still some criticism about the clinical specificity of auricular points/areas representing organs or structures of the body, some researchers have reported scientific evidence of the human body’s somatotopic representation on the outer ear. Romoli et al. (2014) have shown that stimulation of the thumb auricular acupoint (TAA) selectively activates the secondary somatosensory area bilaterally, and that stimulation of the brainstem auricular acupoint (BSAA) mostly activates the cortical and limbic regions that are part of the pain matrix. The results of this pilot study show that the specificity of auricular acupoints can be assessed by functional magnetic resonance imaging (fMRI), and that the brain responses of the two tested acupoints might be linked to their respective therapeutic indications [68,69].

Auriculotherapy has been shown to be effective in reducing chronic musculoskeletal pain [70], stress, anxiety and depression [71], as well as in managing nausea and vomiting in pregnancy [72]. Auriculotherapy has positive effects associated with the conventional treatments of insomnia and chronic and acute pain. Further well-designed studies are required to evaluate this technique’s effectiveness in treating other health conditions [69].

#### 3.1.1. Acupoints Main Characteristics

**Figure 7:**
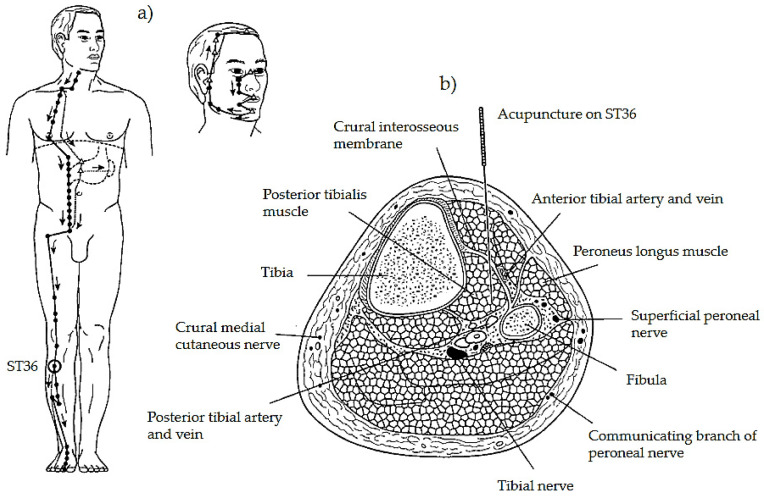
Representation of the stomach meridian and the location of ST36 (**a**). Cross-section of the lower limb at ST36, showing the anatomical structures and a needle puncturing the acupoint (**b**). Reprinted with permission from ref. [64]. Copyright 2002 Elsevier (Amsterdam, The Netherlands) and Copyright Clearance Center (Danvers, MA, USA).

Acupuncture points are often characterized as a composite including blood and lymph vessels and nerves of various types, located within a sheet of loose connective tissue (mesenchyme) perforating the superficial fascia separating subcutaneous tissue from muscle (Figure 7) [73,74,75]. A higher density of gap junctions has been found at the sites coincident with acupuncture points. These gap junctions are hexagonal protein complexes that form channels between adjacent cells, facilitating intercellular communication and increasing electric conductivity [76]. Deep connective tissue structures in locations corresponding to acupoints have been characterized by a higher concentration of Ca, P, K, Fe, Zn, and Mn [75]. It is well known that both Ca^2+^ and K^+^ are critical signal mediators playing an essential role in various physiological activities [77].

For many years, researchers have claimed that acupuncture points are unique locations in the body’s surface, at which the skin’s electrical conductivity is higher than in the neighboring tissue [78,79,80,81,82]. It is also claimed that the conductivity between two points along the same meridian (or conduit) is greater than that between points not sharing this relationship, leading to the commonly held opinion that acupuncture structures are special conduits for electric signals [80]. These findings have led to the theory that the meridians reflect the pathways of least electric resistance throughout the body. Structurally, these may represent fascial cleavage planes, where extracellular ionic fluids can spread electric potentials over great distances without overcoming cellular membranes’ resistances [83]. It is even conceivable that these low-resistance extracellular fluid pathways might have branches connecting them to the internal organs, thus providing some concrete, albeit hypothetical, basis for the traditional organ–meridian associations [64]. These low-resistance fluid channels, where chemical and physical transports occur, are known as low hydraulic resistance channels [84]. The conduit network has also been related to the controversial existence of a primo vascular system known as Bonghan corpuscles and ducts. These thread-like vessels are thought to be similar to blood and lymph capillaries, but are distinct in structure, and some are located inside blood and lymph vessels. Thus, tendinomuscular structures, primo-vessels (Bonghan ducts), and regions of increased temperature and low skin resistance have been suggested as features of the meridian network, or used as identification methods [73].

The analysis of gross anatomic sections of the human arm revealed that about 80% (*p* < 0.001) of acupuncture sites are located in intermuscular or intramuscular connective tissue [85]. Some studies suggest a relationship between the anatomical direction of collagen fibers along the conduits and acupoints, and these structures’ functions [75,86]. Collagen is the main structural protein in the connective tissue, forming a fibrillary matrix containing an interspersed and ordered network of hydrogen-bonded water molecules that supports protons’ rapid conduction, thus acting as a semiconductor [74].

#### 3.1.2. Physiological Effects and Mechanisms of Acupuncture According to Western Medicine

How the process of pain relief is accomplished through acupuncture is not clear-cut, but many science-based theories do exist. The neurophysiological mechanisms by which acupuncture exerts its effects are complex and still under debate. From a Western perspective, acupuncture can be characterized by its effects on the central and peripheral nervous systems. The first one integrates complex somatosensory and cognitive stimulus. When acupuncture acts on the autonomous or vegetative nervous system, it enhances parasympathetic (or reduced sympathetic) activity, which may decrease stress responses and promote immunological homeostasis through the altered brainstem and hypothalamic–neuroendocrine function. Increased vagal stimulation by acupuncture may also initiate the fast “neural” and slow “diffusible” components of the cholinergic anti-inflammatory pathway. In general, the cholinergic anti-inflammatory pathway is driven by brainstem and hypothalamic activity, which may downregulate macrophage activation and suppress the synthesis of tumor necrosis factor (TNF) and other peripheral pro-inflammatory cytokines. Although more research remains to be done, this pathway may play a role in acupuncture efficacy [19].

**Figure 8:**
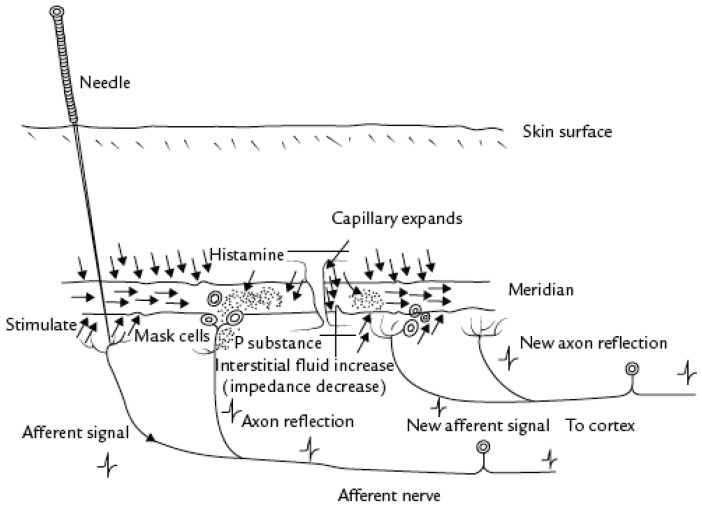
Proposed mechanism for the effect of acupuncture along meridians. Reprinted with permission from ref. [86]. Copyright 2010 Elsevier (Amsterdam, The Netherlands) and Copyright Clearance Center (Danvers, MA, USA).

Following a physiological explanation, the neural hypothesis could be summarized as follows: Acupuncture stimulates small-diameter nerves in muscles, which send impulses to the spinal cord. Then, three neural centers (spinal cord, midbrain, and pituitary) are activated to release specific chemicals (endorphins and monoamines), which act as transmitters blocking “pain” messages (Figure 8).

#### 3.1.3. Effectiveness of Acupuncture

How could a needle inserted in the hand possibly relieve a toothache? Because such phenomena do not conform to accepted physiological concepts, scientists were puzzled and skeptical. Many explained it by the well-known placebo effect, which works through suggestion, distraction, or even hypnosis. However, how does one explain the use of acupuncture analgesia in veterinary medicine if most of the animals are not suggestible? Over the last few decades, researchers have been performing controlled experiments to rule out placebo effects and spontaneous remissions. These experiments have been carried out in clinical practice on patients with chronic pain and by studying acute laboratory-induced pain in humans and animals. From these studies, it can be concluded that acupuncture is more effective than placebo [87].

The positive effects of acupuncture have been reported in treating various conditions [60,63,88,89,90,91,92,93,94,95,96,97,98,99,100,101,102,103,104,105,106,107,108,109,110,111,112]. Indeed, the WHO published in 2002 a detailed review and analysis of randomized controlled trials formally published throughout 1998 (and early 1999) in which acupuncture was proven to be an effective treatment [113]. In 2017, an extensive revision focused on systematic reviews and meta-analyses (the highest form of evidence available) of acupuncture was authored by John McDonald and Stephen Janz. These authors used “The Australian Department of Veterans’ Affairs 2010 Alternative Therapies Review” and the “United States Department of Veterans Affairs Acupuncture Evidence Map 2014” as a starting point. They updated them in a single document known as “The Acupuncture Evidence Project: A Comparative Literature Review”, reviewing the effectiveness of acupuncture for 122 treatments over 14 clinical areas. The authors found evidence for acupuncture’s effectiveness in 117 conditions, with more robust evidence for some conditions than others. Stronger evidence of the positive effects of acupuncture was found for migraine prophylaxis, headache, chronic low back pain, allergic rhinitis, knee osteoarthritis, chemotherapy-induced nausea and vomiting, postoperative nausea and vomiting, and postoperative pain [114].

Acupuncture is best recognized for its use in treating bodily pain. Vickers et al. (2012) conducted a systematic review to identify randomized controlled trials of acupuncture for chronic pain, and found that acupuncture was superior to both sham and non-acupuncture control for each pain condition (*p* < 0.001 for all comparisons). Supported by the significant differences between true and sham acupuncture, these authors concluded that acupuncture has positive effects on chronic pain, and that this therapy must be more than a placebo [96].

Neuropathic pain comes from damage or disease in the somatosensory system. Patients suffering from neuropathic conditions such as trigeminal neuralgia, carpal tunnel syndrome and ulnar tunnel syndrome often report decreased life quality. Carpal tunnel syndrome is a painful and disabling condition affecting the hands when the median nerve, which runs from the forearm into the hand, becomes compressed or inflamed at the wrist. If, one the one hand, the literature exploring the effects of acupuncture in the treatment of symptoms associated with carpal tunnel syndrome point to beneficial effects similar to, or sometimes better than, conventional treatment [115,116], on the other hand, some authors point to little or no effect compared to controls [117,118]. More studies are needed to clarify the ambiguities. Nerve conduction studies (NCS) could be an excellent strategy to assess nerves’ measurable changes after acupuncture. As shown by Chan et al. (2018), while evaluating the effects of acupuncture and moxibustion on the electrophysiological properties of the ulnar nerve, the stimulation of HT4 increased the electrical sensitivity and decreased the stimulus intensity to achieve the maximum amplitude, and avoided a significant increase in latency and decrease in reaction velocity in two consecutive electrical stimulations [119]. Using the same assessment strategy on diabetic peripheral neuropathy (DPN) in type 2 diabetes patients, Meyer et al. (2020) found that classical needle acupuncture had significant effects on DPN, and that improvements in NCS values presumably indicate structural neuroregeneration following acupuncture [120].

Fibromyalgia syndrome (FMS) is a rheumatic disorder characterized by chronic, generalized and diffuse musculoskeletal pain. This disease is more frequent in women than men, and the treatment interventions aim to provide the individual with some pain relief and to restore functionality. Acupuncture has shown positive effects in this condition, with a significant reduction in pain threshold and sensitivity, and improved anxiety, depression and quality of life [89,92,121,122].

Low back pain and shoulder pain are two musculoskeletal conditions frequently treated in daily clinical practice. Low back pain (LBP) is one of the most frequently reported complaints. Several studies point to acupuncture’s effectiveness in treating chronic spinal pain, which, besides low back pain, includes cervical and sciatic pain [95,123,124]. In this field, Molsberger et al. (2002) performed a large, methodologically rigorous double-blinded trial to assess two questions: Can acupuncture contribute as an adjunctive therapy to the conservative management of LBP? Is real acupuncture superior to sham acupuncture? These authors found a pain reduction of 50% or more at three months after treatment. The rates of achievement of the primary outcome, by group, based on intention-to-treat analysis, were as follows: real acupuncture 76.6%, sham acupuncture 29.3%, and conservative orthopedic therapy 13.9% [93].

Additionally, for low back pain patients with pain persisting over more than five years, the relative probability of experiencing 50% or more significant pain reduction was ten times higher in real acupuncture than in sham [93]. In 2010, the same authors conducted a randomized pragmatic, controlled, patient-blinded, multi-center trial to study the effects of acupuncture on chronic shoulder pain. They found significant results for verum over sham and verum over conventional orthopedic treatment (*p* < 0.01). Moreover, the descriptive statistics showed a more significant improvement of shoulder mobility (abduction and arm-above-head test) for the verum group versus the control group immediately after treatment and after three months, indicating that Chinese acupuncture is an effective alternative to conventional orthopedic treatment [94]. These results agree with other studies regarding frozen shoulder [125].

Depression, anxiety and mania are psychopathologic conditions sharing disturbance in mood as a hallmark. These conditions are related to the affection of “shen” and a breakdown of the “yin” and “yang” balance. A patient with depression would be in a state of excessive “yin”, whereas a patient with mania would have excessive “yang”. The reestablishment of a “yin” and “yang” balance might lead to recovery from illness [20]. In this field, acupuncture has shown beneficial results as the primary therapy for reducing the severity of depression [101,102,103,126,127]. Chan et al. (2015) concluded that combined acupuncture and antidepressant treatment is more effective than antidepressants alone in the first six weeks of treatment. Acupuncture can be an effective, safe, and well-tolerated therapy in the early onset of depression, and may reduce antidepressants’ side effects [100].

In the gynecological field, acupuncture helps treat dysmenorrhea, female infertility, and menopausal hot flashes [87,128,129,130]. Often, these conditions induce nausea and vomiting. The stimulation of the acupoints PC6 and ST36 to reduce nausea and vomiting is well documented, and has strong efficacy evidence [131,132]. The proposed action mechanisms stand on the neural response at the insula, hypothalamus and cerebellum responsible for the autonomic regulation of vestibular function, and on the somato-parasympathetic reflex, with improves gastric emptying through increased vagal activity [133].

It is noteworthy that major effects of acupuncture have now also been confirmed by prospective randomized and even double-blinded clinical trials [120,134,135,136,137].

### 3.2. Moxibustion

Moxibustion (“jiu” or “ai”) is based on burning tinder made of Chinese mugwort (Artemisia argyi or Artemisia vulgaris) next to a locus or on it [59]. Artemisia tinder has come to be known in the West as moxa, a Japanese derivation word (“mogusa”, herb for burning). The classical method of performing moxibustion is to make the tinder into a cone and apply it to the skin at points identical to those used for acupuncture. It could be used as a counter-irritant by blistering and scaring the skin, or as a milder form of heat treatment by applying it to the skin with a layer of vegetable material or salt interposed between the skin and the cone. Another method is to combine moxibustion with acupuncture by placing a piece of moxa on top of a needle inserted into the body and igniting it. Thus, the moxa’s heat is conducted down the needle to the surrounding tissues [138].

The physiological changes produced by moxibustion are often associated with the combined action of temperature, radiation, and the pharmacological effects of burning Artemisia and its combustion products [139,140]. The warm temperature of moxibustion induces antipyretic and thermolytic effects by stimulating polymodal receptors in the skin at zones corresponding to acupoints. Vasoconstriction in the treated point and vasodilatation around it are often experienced with increased peripheral blood flow and microvascular permeability. In addition, heat shock proteins naturally synthesized in cells in response to hyperthermia might be induced in local tissues due to the increased temperature. Nonthermal effects are associated with the visible light and infrared radiation emitted by burning moxa and absorbed by the connective tissue, blood and lymphatic vessels, and nerves, which might induce some active substances such as cytochrome c oxidase and intracellular water, two photoacceptor molecules or chromophores. Changes in the water dynamics in membranes, mitochondria and/or cells could modulate signaling pathways, the production of reactive oxygen species (ROS), ATP (adenosine triphosphate), Ca^2+^, NO, and inositol phosphates group, with effects on stress signaling, metabolic processes, cytoskeleton organization, cell proliferation/differentiation, and homeostasis [141]. The photoelectric effect and photochemical process generate energy that might help adjust the body’s immune and neurological functions.

The major subproduct of burning moxa is smoke, and its security is still under debate. Studies using solid-phase microextraction gas chromatography–mass spectrometry (SPME-GC-MS) have shown that moxa smoke is composed of furan-structure substances, aromatic compounds, esters, alkanes, and hydroxyl-containing compounds [135]. Although these compounds’ toxicity represents a concern, moxibustion has been considered minimally toxic, safe and effective, with few adverse events [142]. Research has shown that no harmful effects of moxa smoke have been noticed on the heart rate (HR) and heart rate variability (HRV) of treated persons. Indeed, Cui et al. (2013) reported decreased HR and increased HRV during 25 min moxa smoke exposure, suggesting that moxa smoke has a regulating effect on the autonomic nervous system, and its inhalation has short-term stress-alleviating effects [143].

Nevertheless, excessive inhalation should be avoided due to the increased incidence of chronic laryngitis, as noticed in five Chinese medicine hospitals in the Guangdong province. A survey of moxibustion practitioners taken in those hospitals showed that moxibustion smoke raised the incidence of chronic laryngitis from 3.70% (nonacupuncturists) to 26.67% (acupuncturists) (*p* < 0.05). Moxibustion safety depends on the position, the duration, the distance between moxa and skin, the practitioners’ proficiency, patient conditions, stimulations from smoke, room conditions, and extraction [142].

Moxibustion has been reported as a successful therapeutic in treating knee osteoarthritis [144]. Research shows that patients treated with moxibustion experience significantly improved pain, stiffness, and physical function compared with sham-moxibustion [145]. Although the majority of systematic reviews and meta-analyses of randomized controlled trials point to absences in the research and request further well-designed, large-scale RCTs, the overall outcome points to positive effects in managing the symptoms of post-stroke urinary incontinence [146] and constipation [147], use as an adjuvant therapy for chronic kidney disease [148], and in treating diabetic peripheral neuropathy [149]. Limited or low evidence of positive effects was found in chronic low back pain, [150] and in treating lumbar disc herniation [151].

### 3.3. Cupping

Cupping is another ancient technique commonly used in TCM. This technique helps the body expel pathogenic factors such as cold or “algor”, dampness or “humor”, and wind or “ventus”, and treats conditions related to the stagnation of “qi” and “xue”, such as bruises or sore muscles. It is beneficial for various pain types in the lower back, shoulders, and legs [152,153]. However, it has also been reported as a successful intervention in both usual and emergency treatments, such as herpes zoster, acute asthma episodes, angina pectoris and abdominal pain induced by poisoning [154,155,156]. Cupping is used to move stagnant blood out of deep bruises by bloodletting, and reduce swelling and pain in sprains. Cupping can be done by heating the air within the cupping glass and then putting the cupping glass on the skin. Cooling the air by clapping the cup over the affected area exerts mild suction. The pull exerted pushes the flesh into the cup, mobilizing body fluids into the area [64]. Simple cupping of a point is believed to suck off “humor”, removing excess fluid from the point. Often, this “humor” comes with “ventus” as “humor venti”. Thus, cupping is also used to treat “ventus” [12]. Cupping can also be performed over the needle to enhance acupuncture effects. From a Western perspective, the cupping action mechanisms are still unclear. The sub-atmospheric pressure inside the cup seems to change the skin’s biomechanical properties, increasing peripheral blood circulation and pain threshold, improving local anaerobic metabolism, reducing inflammation, and modulating the cellular immune system [157]. The comfort and relaxation sensation on a systemic level often reported after cupping might be related to the resulting increase in endogenous opioid production in the brain leading, to improved pain control [158].

## 4. Chinese Manual Therapy (“Tuina”)

Although the origins of Chinese bodywork predate written records, written sources from the Qin Dynasty in the third century BC refer to manual therapy as “Moshou” (hand rubbing). The term “manual therapy” indicates treatment where the hands are used as the primary intervention tool. A century later (Han Dynasty, 206 BC–221 CE), it was called “Anmo” (press and rub), a term still used today. Palpatory techniques for diagnosis and manual treatment techniques are described in the “Huangdi Neijing” [64]. This art was developed, improved and spread over time by the Chinese laborer people in their continuous fight against diseases. Many aspects of “Tuina” have come from the martial arts lineage. Here, the bodywork was used to heal traumatic injury and correct structural misalignments, and keep the martial artist fit and healthy [159]. “Tuina” can affect the five sense organs’ health and help a person feel more vivid via hetero or auto treatments, wherein specific self-massage applications play an essential role [64].

**Figure 9:**
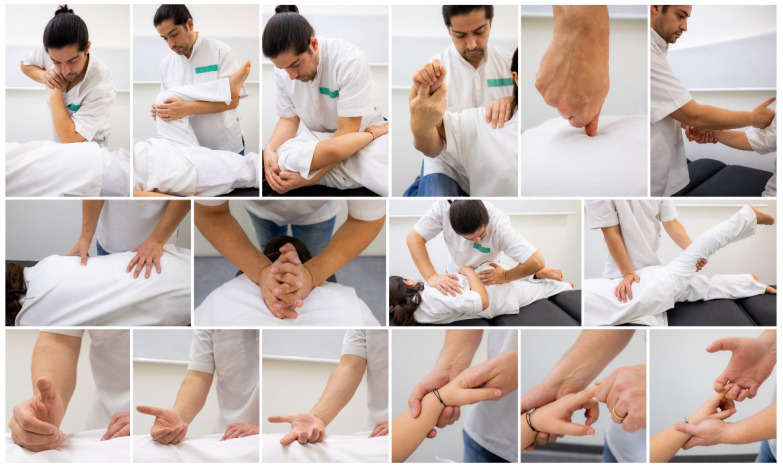
Examples of “Tuina” techniques used to treat various complaints.

“Tuina” (push and pull) and “Anmo” (press and rub) refers to a system of massage, manual acupoint stimulation, and structural manipulation performed by a fully trained practitioner, who combines skills that in the West would usually be divided into massage, physical therapy, osteopathy and chiropractic [160]. A practitioner may also know and incorporate Chinese herbs, plasters, bone setting, and “qi” projection. Originally, “Tuina” was developed to treat traumatic injury and for use in pediatric care, whereas “Anmo” was directed toward the treatment of internal diseases [64]. A distinct aspect of “Tuina” is the extensive training of the hands necessary for clinical practice. The practitioner’s hands are trained to accomplish focused and forceful movements, applied to several body areas. Techniques such as pushing, rolling, kneading, rubbing, and grasping are practiced until they become second nature (Figure 9). Students practice on a small bag full of rice until their hands develop the necessary strength and dexterity. “Tuina” is often applied to limited body areas, and the techniques can be quite forceful and intense [160].

“Tuina” is known as “outer therapy”, and like acupuncture and other techniques, acts on acupoints and structures of the skin. It integrates a vast number of techniques acting on stimulating specific points, muscles and connective tissue, and triggering the reflexes. A typical intervention has three phases:

1. In the activation phase, the meridian is stimulated to remove the build-up more easily;
2. In the intervention phase, the removal of the build-up is performed by specific diagnosis-related techniques;
3. In the harmonization phase, the intervention’s strong effect is normalized with the surrounding tissue’s physiological proportions, and is therefore “harmonized”.

“Tuina” manipulation is experience-dependent, and its therapeutic efficacy is influenced by many operating variables, mainly frequency, duration, and force, which make the standardization process difficult. Research to identify the standard features for “Tuina” manipulation should consider the muscle groups involved and their potential cooperation, the motion angle, and the joint forces during manipulation. It is also essential to quantify the frequency, duration and applied force’s biological effects. Even so, based on the available literature, the most standardized “Tuina” manipulations seem to be the ones involving minimum consumption but achieving maximum therapeutic benefits [161].

“Tuina” is routinely used in patients with orthopedic and neurological conditions and for treating joint and injury problems, chronic conditions, and back problems [162,163]. Because these techniques can affect the functioning of the body’s internal organs, they are appropriate for internal medicine, gynecology and trauma, and for patients with conditions that may not be thought of as susceptible to treatment through manipulation. The previous may include asthma, dysmenorrhea, chronic gastritis, hypertension, failure to thrive in preterm infants, major depressive disorder, substance abuse and dependence, pain syndromes, and immune and autoimmune conditions [164,165,166,167,168,169].

“Tuina” is an adjunct to acupuncture, used to increase the range of motion of a joint, or instead of acupuncture when needles are uncomfortable or inappropriate, such as in pediatric applications [160]. In this field, Wang et al. (2012) conducted a study to evaluate the impact of “Tuina” in treating pediatric muscular torticollis [170]. Of the 38 treated cases, the authors reported 34 cases as full recovery, 3 cases as remarkable effectiveness, 1 case as effective and 0 cases as ineffective. With a 100% total effective rate, they concluded that the combined method for treating muscular torticollis regulated tendons, thus relieving blood stasis, and it also improved muscular spasms, dispersed mass, and enhanced and restored neck function [170]. “Tuina” combined with medicinal decoctions has shown positive therapeutic efficacy in treating pediatric allergic rhinitis [167]. As a standalone technique, “Tuina” seems to reduce diarrhea frequency in children aged 0–6 years, compared with sham [171].

## 5. “Qigong”

The term “Qigong” is composed of two words: “Qi”, which was previously explained and could be understood as vital energy or, according to some authors, ethereal dynamic energy with feedback potential [172], and “gong”, which means the development of capacity. Therefore, “Qigong” is a practice that allows the development of the capacity to collect, circulate and apply vital energy.

“Qigong” integrates three primary schools: medical, martial and spiritual. Although they differ in purpose, they are based on the same philosophical system, sharing several techniques. In essence, medical “Qigong” promotes health, longevity, and the prevention, diagnosis and treatment of diseases and imbalances. Martial “Qigong” focuses on developing the strength and power of martial artists, and spiritual “Qigong” searches for spiritual enlightenment and transformation.

The main therapeutic goals of “Qigong” are [6,173]:

1. To eliminate internal pathogenic factors (excessive accumulation of emotions such as anger, sadness, fear, worry) as well as external pathogenic factors (cold or “algor”, heat or “calor”, dampness);
2. To harmonize the “qi” flow, promoting orthopaty (self-healing power) and avoiding inauspicious depletion (lack of activity) and repletion (excess of activity);
3. To regulate and balance the patient’s “yin yang” functional status, so as to restore harmony.

As a therapeutic tool, “Qigong” can be used as a self-regulation practice or a mediated healing intervention. In the first case, several exercises can be prescribed according to the person’s condition, and then performed with the required periodicity. In the second case, the practitioner uses the so-called “qi emission” techniques to restore balance in the patient. Distance therapy (also called “qi” emission or external “qi”) requires the “Qigong” practitioner to manipulate the patient’s “qi” by focusing on the energetic properties of the patients’ channels, collaterals, and points, as well as internal organs, from a distance of several inches, several feet, or even several miles away [6].

### 5.1. “Qigong” and the Defensive “Qi”

TCM theory considers that the human body generates an external defensive field known as “wei qi”. According to the Heidelberg model of TCM, the defensive “qi” is located outside the conduits, within the tissue. It has its origin in the three functional sections of the body (three burners or “calorics”), and centers on, predominantly, the surface (“extima”), where the pulmonary orb ensures its distribution [7,10,13,173]. This “wei qi” field is believed to include three external layers of subtle energy (physical, emotional and spiritual), each one connected to one of the three “dantians” and surrounding the physical body.

The external “wei qi” field protects the body against external pathogenic factors’ incursions, interacting with the surrounding environmental energetic fields. These may include geomagnetic rhythms, Schumann resonance, shallow frequency electromagnetic radiation, X-rays, cosmic rays, as well as the radiation provided by our technology [174,175,176,177,178]. Both external and internal pathogenic agents affect the “wei qi” field’s structural formation. Internal factors include repressed emotions such as anger and pain from emotional traumas. Strong non-processed emotions block the regular circulation of “qi”, creating stagnation in the body. The external factors include chronic and severe environmental agents, such as coldness, humidity, dampness, heat and wind. Physical traumas also affect the “wei qi” field by creating fragilities in the external energetic matrix. These fragilities create vulnerabilities, making it easier for disease-causing pathogens to enter.

During “Qigong” practice, the “qi” is captured and absorbed in the lower “Dantian”. The practitioner should idealize and feel this energy extending into the Earth (as an anchor), and the surrounding “wei qi” field increasing and expanding [6].

### 5.2. “Qigong” as Traditional Biofeedback Therapy

**Figure 10:**
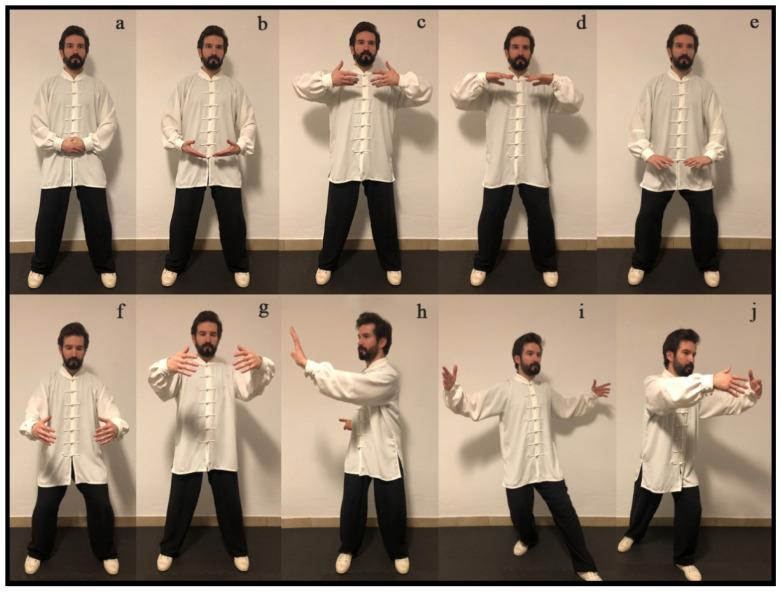
“Qigong” exercises. Sequence (**a**–**g**): (**a**) focus on the lower “dantian”; (**b**–**e**) moving the “qi”; (**f**) white ball lower “dantian”; (**g**) white ball middle “dantian”. Sequence (**h**–**j**): (**h**) “Taiji Qigong” pushing palm; (**i**,**j**) “Taiji Qigong” spreading the wings.

“Qigong” practice promotes the development and control of “qi”, and its balanced distribution through the body. “Qigong” is considered a traditional vegetative biofeedback therapy that uses postures, movements, and breathing exercises combined with meditation to induce the vegetative stabilization and self-regulation of the body’s biologic systems. The “qi activation” is achieved by breath control and a particular mental state of “awareness” [179,180], thereby improving and strengthening the overall state of vegetative regulation (homeostasis) [181,182,183,184,185]. The “Qigong” practice consists of specific techniques (Figure 10) that use knowledge of the body’s internal and external energy fields to purge, invigorate, and balance these energies. Medical “Qigong” therapy offers patients a safe and effective way to rid themselves of pathogenic agents, such as painful emotions that otherwise can cause mental and physical illness. As mentioned above, this therapy combines breathing techniques with movement, creative visualization, spiritual intent to improve health, personal power, and control [6].

Breathing control is particularly important in “Qigong” practice. The breath is the most important source of “qi”, related to the Metal Phase (rhythmical distribution of energy) and the pulmonary “orb”. Breathing is the pacemaker of several vegetative functions, such as the muscular tonus and the capillary blood flow. “Qigong” breathing exercises (“Tu Gu Na Xin”—“expel the old and absorb the new”) promote the capture and absorption of “qi” from the air, increasing the body’s vitality and the harmony between “qi” and “xue”, while promoting health and purging disease [6].

As part of the “orbs”, breathing patterns are directly connected to the five primary emotions significantly affecting the “qi” circulation. Breathing is a connection between mind and body, with effects on physiological functions and emotional balance. Anger or “Ira”, which belongs to the Wood Phase, increases the “qi”, making the exhalation more vigorous than the inhalation. Sadness, grief and pain (“maeror”), which belong to the Metal Phase, deplete the “qi”, making the inhalation more substantial than the exhalation. Fear and shock (“pavor”), which belong to the Water Phase, decrease and disperse the “qi”, inducing a short and superficial breath, as a result of the kidney’s incapacity to retain the “qi”. Excitation and luxury (“voluptas”), which belong to the Fire Phase, induce irregular breathing with fast-shifting patterns. Worry (“solicitude”) and reflection (“cogitation”), which belong to the Earth Phase, block the “qi” and make the inhalation short and weak, which is sometimes sustained during a period, followed by fast inhalation and exhalation [6,173].

### 5.3. Physiological Effects and Mechanisms of “Qigong” Practice

One of the prime benefits of “Qigong” is stress reduction, and one of the main concepts of this practice is to use the mind to guide activation and deactivation patterns by imagination. Excessive stress may negatively impact a person’s health state and may be associated with increased anxiety, psychological disorders, and functional impairments of the organs within the body [186]. It has been shown that “Qigong” and “Taijiquan” training may reduce emotional exhaustion, depersonalization, and even improves anxiety, and reinforce attention and effectiveness in high school students [187,188,189,190,191,192,193].

The “White Ball” is one specific type of “Qigong” similar to the “Zhan Zhuang” system. Children quickly learn this system when enrolled in a formal training program, with evident development of their individual vegetative skills and reduced anxiety-induced effects [187,188,194]. Research has shown that these skills are stable after weeks of training, allowing young musicians to play the flute in auditions, with warm fingers and reduced anxiety-induced elevation of heart rate, even without a momentary prior application of the learned “Qigong” exercises. It seems that positive vegetative changes in the behavioral pattern appear to be naturally available on-demand in critical stressful situations as a part of the child’s reactive behavioral repertoire [187].

**Figure 11:**
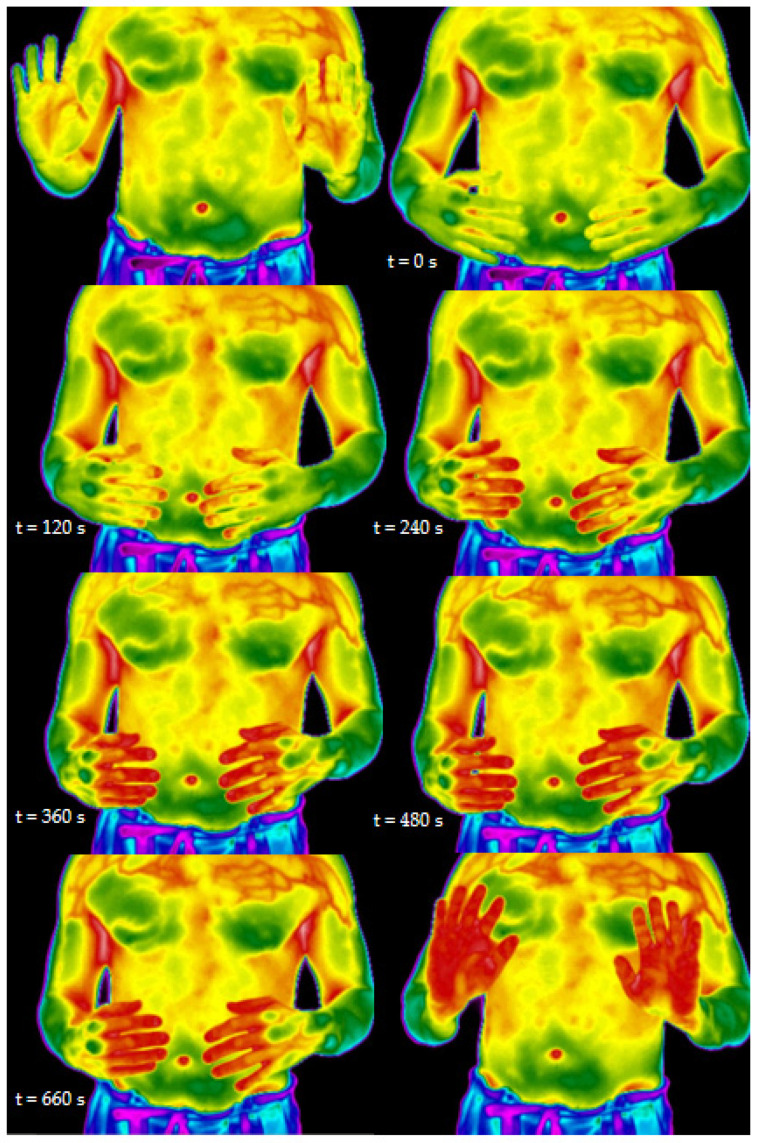
Thermograms of the “Qigong” exercise known as “White Ball”. Reprinted with permission from ref. [197]. Copyright 2019 Elsevier (Amsterdam, The Netherlands) and Copyright Clearance Center (Danvers, MA, USA).

Infrared thermography can be used to measure the dynamic changes of temperature in the hands during “Qigong” practice (Figure 11). This technique has been reported while studying “Qigong”-related effects [195,196].

Thermography measurement showed that the “Qigong” exercise could change the fingers’ temperature. Therefore, skin temperature changes may be interpreted as an increase in microcirculation. Researchers found that when a particular mental state of awareness was achieved, and the “qi” sensation was felt, skin temperature increased to 37 °C [194]. These data may also help demystify Chinese medicine, while thermography allows for visualizing the microcirculation effects on the hand’s temperature during “Qigong” practice. TCM holds that the “mind” guides the “qi”, which therefore guides the “xue” (“blood”). The Heidelberg model of TCM sees strong analogies between the effects of “xue”, as described by the classical scriptures, and the clinical effects of microcirculation in Western medicine. Therefore, “qi”, translated by this model as a vegetative functional capacity, guides and steers microcirculation. In other words, this old phrase from classical scriptures can be demystified accordingly: “the mind” (imagination and awareness) can guide and therefore activate vegetative capacities, which in return lead to changes in the microcirculation [187].

The effects of “Qigong” and meditation on brain activity self-regulation through biofeedback have been reported. Research has explored brain activity biofeedback with real-time functional resonance magnetic imaging (fMRI) during pain stimuli, and has found that subjects could learn to control activation in the rostral anterior cingulate cortex (rACC). Researchers found a deliberate increase or decrease in fMRI signal in the rACC, corresponding to a change in the perception of a thermal pain stimulus. Furthermore, chronic pain patients could be trained to control activation in rACC, thereby decreasing their ongoing chronic pain level. Meditation has also been explored to control clinical pain. It has been shown that long-term meditators have a diminished thalamic response to experimental pain stimuli compared with age-matched non-meditators. Meditation involves a state of altered consciousness and may increase alpha wave power in occipital, parietal and temporal brain regions, as well as gamma power, as measured by magnetoencephalography (MEG) and electroencephalography (EEG), respectively. Long-term meditation practices may also help preserve the brain’s regions, including the prefrontal cortex and right anterior insula [67].

Research has shown that the effectiveness of “Qigong” in balancing the spirit (“shen”) is remarkable. Tsang et al. (2006) studied the effect of “Qigong” practice on the psychosocial behavior of depressed elderly individuals, and concluded that regular “Qigong” practice could relieve depression and improve personal efficacy and well-being [184]. Furthermore, in the field of psycho-emotional imbalances, Griffith et al. (2008) found a significant reduction in stress while studying the efficiency of a “Qigong” training program on hospital staff’s stress management [190]. Saganha et al. (2012) obtained similar positive results after a three-week “Qigong” training program for physiotherapists suffering from burnout. In the previous study, the program’s efficacy was accessed by the Maslach burnout inventory (MBI) questionnaire, and the results show that the program was able to decrease the mean values of emotional exhaustion and lower the mean values of depersonalization [189].

Furthermore, in autism, some positive effects could be noticed, as shown in the study conducted by Silva et al. (2005). These authors found that a group of eight children aged below six years, regularly submitted to “Qigong” massage, had decreased autism behavior, improving speech, sensory and motor skills, and general well-being [198].

A considerable number of studies point to significant changes in physiological parameters, such as blood pressure and circulation, heart rate and variability, plasma triglycerides, total cholesterol and low-density lipoprotein (LDL) cholesterol, HDL cholesterol, skin temperature, lung functions (such as the increment in forced expiratory volume and a reduction in the number of exacerbations), relaxation state measured by electroencephalography, light emission measured by photon counting, electrical charge measured by gas discharge visualization, and electrical conductance and the potential of acupoints [194,196,197,199,200,201,202,203,204,205].

Hypertension is a worldwide transversal concern, especially in the elderly population, as aging may decrease the blood vessels’ elasticity and increase the risk of other hypertension-related comorbidities, such as obesity, diabetes and kidney disease. Lee et al. (2003) found that “Qigong” promotes the relaxation and stabilization of the sympathetic nervous system in hypertension patients, positively modeling urinary catecholamine levels and blood pressure, and improving ventilatory functions [180]. Recent systematic reviews on the subject confirm these results [206,207]. Controlled studies developed by Lan et al. (2004) to access the cardioventilatory behavior of elderly “Qigong” and “Taijiquan” practitioners showed an improvement in aerobic capacity [185]. “Qigong” also seems to benefit the overall quality of life of older adults with chronic diseases [208].

Other common diseases treated by “Qigong” include diabetes, arthritis, hypertension, breast and ovary cysts and tumors, migraine, fibromyalgia, insomnia, acute abdominal pain, colitis, muscular atrophy, brain tumors, stroke, coma recovery, and certain types of cancer [179,180,181,182,183,186,209,210].

## 6. Conclusions

Over the last few decades, Western culture has changed its views on healthcare, with a continuous shift in tendencies and an increasing demand for complementary and alternative medicines. These changes could be supported by the easier availability of these health practices, and people’s beliefs, convictions and preferences have also been changing. Within this new paradigm, Western health systems tend to adapt themselves to the population’s demands.

Western medicine is based on mainstream science, and, therefore, the standard approach to CAM is the integrative way. This process requires a profound evaluation of effectiveness and security, which has an inherent science-based conceptualization and standardization effort that goes from diagnosis to therapeutics. Even so, the articulation of these two paradigms, conventional and traditional, must be balanced, as balanced as the relation between “yin” and “yang”, never denying the power of cultural, philosophical foundations, and taking into consideration millennia of empirical evolution and created knowledge.

In this line of thought, the integration of TCM into Western health systems and research requires a pragmatic science-based approach. The standardization of diagnosis and therapeutic methodologies is a crucial factor. This process requires measuring a wide range of variables in a representative sample and statistical procedures to validate the findings. As shown in a previous review, this can also lead to new technological systems to measure TCM-related effects, and to calibrate and mimic complex and skillful diagnosis procedures [211]. A second crucial factor is the further development of experimental approaches in TCM research, such as the double-blinding of acupuncture trials [120,134,135,136,137] and the development of a placebo for “Qigong” research [212].

TCM therapeutic success is closely dependent on the accuracy of the diagnosis. TCM diagnosis is an intricate art dealing with evaluating a matrix of variables, some of them subtle and dependent on the practitioner’s experience and sensibility. Generally, the diagnosis involves looking, listening, palpating and questioning, to establish a general picture of the patient’s condition, involving subtle and physical factors related to their inner nature, their pathological activity and their physiological status. By looking at the patient, the practitioner evaluates the brightness of the eyes, the facial complexion, the body structure and motion, the skin, the hair and the tongue. Listening involves listening to the patient’s breathing, the characteristics of the voice and coherence of the speech, and palpating involves palpating the pulse at the radial arteries on each wrist and pressing the alarm points in the body. Examples of questioning involve asking the patient about causes, onset, duration, symptoms, previous treatment results, appetite, diet, energy, memory, mood, liquid and solid excretions, and sleep. Although some of the previous variables seem to be easy to gauge and common to Western medical diagnosis, others, such as TCM pulse diagnosis, are complex, skill-dependent, and based on TCM physiological concepts. Pulse diagnosis involves feeling the pulse at the radial arteries of both wrists, in three gauging sites, at two or three depths, individually or simultaneously, depending on the practitioner’s training and experience. The diversity of pulse qualities felt by the practitioner includes, but is not limited to, replete, moderate, weak, floating, stirred, intermittent, racing, sunken, dissipated, hollow, slow, rapid, surging, fine, vacuous, long, short, slippery, rough, string-like, tight, soggy, faint, drum skin, firm, bound, skipping and hidden. This represents an obvious challenge in research, as mainstream science demands the accurate parameterization and standardization of procedures and protocols to promote reproducibility and validate results.

TCM standardization requires a rational communicable theory and language that translates to Western physiology the structural concepts of TCM, making possible the inherent rational use of the reflex therapeutic systems, anti-inflammatory mechanisms, and mental training involved, for example, in acupuncture and “Qigong”. This is of primary importance to overcome the ambiguities and vagueness that some Western healthcare practitioners and researchers experience when dealing with the philosophical framework and structural concepts of TCM, such as the “yin yang” and the “qi”. The inclusion of a supplementary chapter on traditional medicine conditions in the International Statistical Classification of Diseases and Related Health Problems (ICD-11) was a significant step in the clarification process. The World Health Organization plays an essential role by establishing criteria, guidelines and strategies, and updating the healthcare community with the evidence-based results pertaining to TCM practices, such as reviewing and listing various diseases or disorders for which acupuncture therapy has been tested in controlled clinical trials.

TCM practices’ security is a critical issue to consider in the Western healthcare integration process. Although most TCM therapeutics, such as acupuncture, “Tuina”, and “Qigong”, are commonly accepted as safe, herb use remains a significant concern, mainly due to its origin, quality and dose-related toxicity. The existence of scientific evidence on efficacy and security, and quality control standards supported by in vivo or in vitro clinical trials, are essential to ensure the quality, toxicity and safety of Chinese herbal medicine. In this process, Chinese herbal medicine’s efficacy must be evaluated via the same standards used in Western medicine. Well-designed randomized controlled trials, considered the highest level of evidence required to establish causal relationships in clinical research, are crucial to breaking down doubts and resistance.

TCM is a challenging area of research. Although some practices defy the dominant biomedical paradigm, the effort being made by the scientific community, and the number of positive results derived from clinical applications studying either its nature and mechanisms, are significant. Moreover, challenging experience is crucial to promoting continuous scientific innovation and breakthroughs.

## Figures and Tables

**Table 1:** Meanings of “yin yang” within the regulatory context (adapted with permission from Greten, H. (2008). Copyright 2008 Heidelberg School of Chinese Medicine [10]).

“Yin”	“Yang”
Bellow the target value	Above the target value
Descending values, such as in downregulation	Uprising values, such as in upregulation
Lack of substrate causing unstable regulation	Functional, primordial regulatory problem

**Table 2:** Meanings of “yin yang” within the medical context (adapted with permission from Greten, H. (2008). Copyright 2008 Heidelberg School of Chinese Medicine [10]).

“Yin”	“Yang”
Less vivid, less “qi” (depletion)	More vivid, more “qi” (repletion)
Colder, “algor”	Warmer, “calor”
Inside, interior, “intima”	Outside, exterior, “extima”
Structure	Function

## Data Availability

Not applicable.
